# Head-to-head comparison of image quality between brain ^18^F-FDG images recorded with a fully digital versus a last-generation analog PET camera

**DOI:** 10.1186/s13550-019-0526-5

**Published:** 2019-07-12

**Authors:** Julien Salvadori, Laetitia Imbert, Mathieu Perrin, Gilles Karcher, Zohra Lamiral, Pierre-Yves Marie, Antoine Verger

**Affiliations:** 1 IADI, INSERM UMR-1254, Université de Lorraine, 54500 Vandœuvre-lès-Nancy, France; 2 Plateforme Nancyclotep, CHRU-Nancy, Université de Lorraine, 54500 Vandœuvre-lès-Nancy, France; 3 Service de Médecine Nucléaire, CHRU-Nancy, Université de Lorraine, 54500 Vandœuvre-lès-Nancy, France; 4 Institut de Cancérologie de Lorraine, Université de Lorraine, 54500 Vandœuvre-lès-Nancy, France; 5 Centre d’Investigations Cliniques Plurithématiques, INSERM UMR-1116, Université de Lorraine, 54500 Vandœuvre-lès-Nancy, France

**Keywords:** Digital PET, Brain ^18^F-FDG, Image quality, Contrast, Spatial resolution, Noise

## Abstract

**Background:**

The quality of phantom images was previously shown to be higher on digital (Vereos Philips®) compared to analog PET (Ingenuity Philips®) cameras. This study aimed to determine the extent to which this difference still remains significant on normal brain ^18^F-FDG PET images.

**Methods:**

Relative noise and contrast as well as border sharpness (a spatial resolution index) of central (striata) and peripheral (occiput) gray-matter structures were compared between 10 sets of normal brain ^18^F-FDG PET images recorded and reconstructed on digital and analog last-generation PET cameras, together with a subjective visual analysis of image quality provided by experienced physicians.

**Results:**

Compared with analog PET, digital PET provided marked improvements in image quality parameters. The median relative noise was decreased (− 22%), while gray/white-matter contrast was increased (+ 27%/+ 41% for central/peripheral gray-matter structures), with these results being consistent with visual analysis. In addition, a clear enhancement in image sharpness was further documented for digital PET owing to the possible use of a 1-mm^3^ voxel size (+ 24%/+ 21%).

**Conclusions:**

On normal brain ^18^F-FDG images and compared with a last-generation analog PET, the fully digital PET camera offers marked improvements in image noise and contrast, as well as significant potential for further enhancing spatial resolution.

**Electronic supplementary material:**

The online version of this article (10.1186/s13550-019-0526-5) contains supplementary material, which is available to authorized users.

## Background

Fully digital PET cameras, such as the “Vereos” (Philips®, Cleveland, Ohio), feature small digital silicon photomultipliers instead of much larger photomultiplier tubes, providing true digital photon counting with 1-to-1 crystal coupling [1]. These properties are primarily likely to enhance the time-of-flight (TOF) capability and thus to favor signal-to-noise ratio [2].

According to the PET standards of the National Electrical Manufacturers Association (NEMA) [3], the Vereos digital PET camera exhibits certain advantages, particularly with regard to time of flight (TOF) resolution (~ 310 ps) leading to an enhanced signal-to-noise ratio gain [2] and ultimately to an improvement in image contrast (contrast recovery ranging from 54–62% for 10-mm hot sphere to 84–88% for 22-mm hot sphere) and noise level (background variability ranging from 8.8–9.6% for 10-mm sphere to 2.5–2.6% for 37-mm sphere) [4, 5]. Spatial resolution is additionally enhanced with this digital PET camera (4.0–4.2 mm FWHM at the field of view center) presumably owing to the 1-to-1 crystal coupling (lower uncertainty in the interaction position) and to the smaller field of view (lower impact of the non-collinearity of coincident photons) [4, 5]. Indeed, fields of view of this digital PET camera are respectively 9% (16.4 vs. 18 cm) and 15% (76.4 vs. 90.3 cm) smaller than those of a last-generation analog PET camera, the “Ingenuity TF” (Philips®, Cleveland, Ohio), in axial and transversal directions.

Previous pilot clinical studies have also led to consider that digital PET might improve not only the image quality of whole-body PET images, but also diagnostic confidence and accuracy for oncologic diseases, as compared with analog PET [6–8]. Such a significant enhancement in image quality and in diagnostic accuracy could prove helpful for various PET exams and particularly for brain imaging where it could additionally facilitate current dual PET/MRI analyses [9]—i.e. with a greater ability to delineate cortical gyri than with analog PET [10]. However, it is not known whether digital PET objectively offers a significant and quantitative gain in the quality of brain PET images.

This study thus aimed to assess the image quality of normal brain ^18^F-FDG images, recorded and reconstructed with the Vereos digital PET camera, as compared with those obtained with a last-generation analog PET camera, the “Ingenuity TF” (Philips®, Cleveland, Ohio).

## Methods

### Study population

Two groups of 10 sets of normal brain ^18^F-FDG PET images, respectively recorded on analog (Ingenuity, Philips®, Cleveland, Ohio) and digital (Vereos, Philips®, Cleveland, Ohio) PET cameras, were selected in our department. All patients had been referred in November or December 2017 as a part of an oncological workup for a whole-body ^18^F-FDG PET exam starting with a brain PET recording, performed 45 min after injection of 3 MBq/kg (81 μCi/kg) of F-18 FDG. The selection was based on the following criteria: (1) absence of any known neurological or psychiatric disease, (2) absence of diabetes mellitus and of a blood glucose level > 2 g.L^−1^ at the time of ^18^F-FDG injection, (3) brain PET and CT images considered definitely normal through a careful visual analysis performed by an experienced observer (AV), and (4) matching between the 2 groups according to the patients’ gender and age (±5 years). This study was approved on April 23, 2018, by the local institutional review board (Comité d'éthique CHRU Nancy). The study was conducted in accordance with the Declaration of Helsinki and all patients from our institution are informed that their medical data can be rendered anonymous and used for scientific purposes.

### PET imaging

The brain PET data from both cameras were recorded in list mode over a 15-min period in a single-bed position. All images were first reconstructed as currently recommended by the manufacturer, namely with 2-mm isotropic voxels, using an ordered subset expectation maximization (OSEM) algorithm with the TOF information and by accounting for dead time losses as well as scatter, random, and attenuation corrections with CT images for both cameras.

Digital PET images were additionally reconstructed with the same method but with a 1-mm voxel size in order to assess the potential for further enhancing spatial resolution. The analog PET device was conversely not equipped with a reconstruction system yielding a voxel size of 1-mm.

**Fig. 1:**
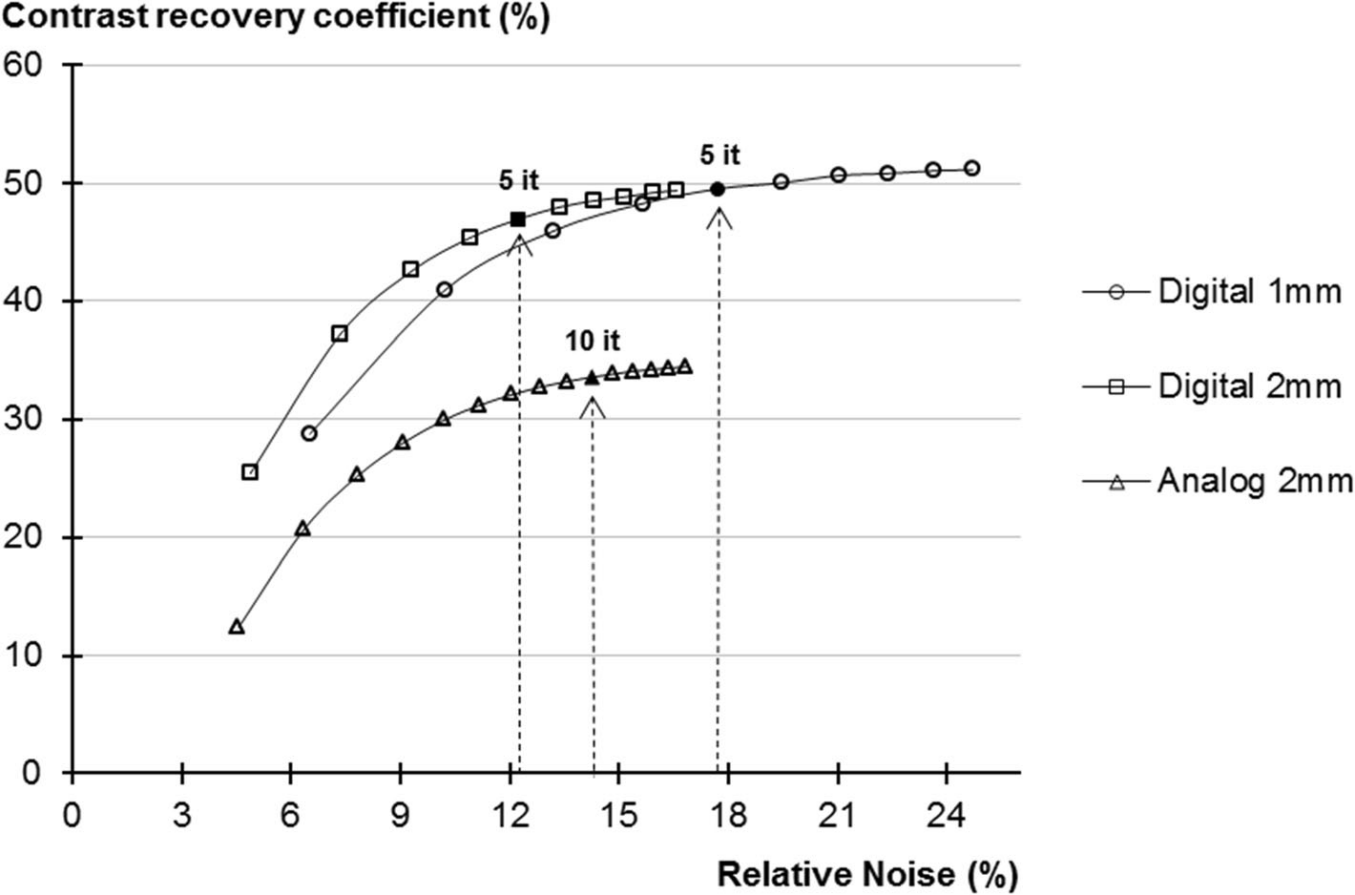
Contrast recovery coefficients determined in percentages for the 10-mm hot sphere, with 2-mm voxel size for analog and digital PET and with 1-mm voxel size for digital PET and displayed according to relative noise (coefficient of variation in the background, in percentages) for each number of iterations. Black symbols and dashed arrows represent the numbers of iterations considered to reach sufficiently high levels of convergence. This convergence was reached at 5 iterations for the digital PET with either 1-mm or 2-mm voxels size and at 10 iterations for the analog PET

In order to determine the number of OSEM iterations allowing the comparison of the two cameras under similar convergence conditions, an IEC torso phantom was performed on both systems following the NEMA NU-2 2012 protocol (i.e. with 5.3 MBq/L in the background and a 4:1 sphere-to-background ratio) and reconstructed with 10 subsets and a number of iterations ranging from 1 to 10 for digital and from 1 to 15 for analog cameras, respectively. The convergence of the contrast recovery coefficient of the smallest 10-mm hot sphere was chosen as a convergence criterion for this study. As detailed in Fig. 1, where contrast recovery coefficients are plotted according to the relative noise (coefficient of variation in the background), this convergence was reached at about 5 iterations on the digital PET with either a 2-mm or 1-mm voxel size and about 10 iterations on the analog PET. Consequently, OSEM reconstructions were performed with 3 iterations and 15 subsets and 3 iterations and 33 subsets, respectively, for digital and analog cameras.

The reconstructed PET images were post-treated both without and with the additional use of a current method of resolution recovery obtained by a deconvolution of the point spread function (PSF) using a regularized version of the Richardson-Lucy algorithm [11, 12] (1 iteration and 6-mm regularization kernel).

### Performance parameters

**Fig. 2:**
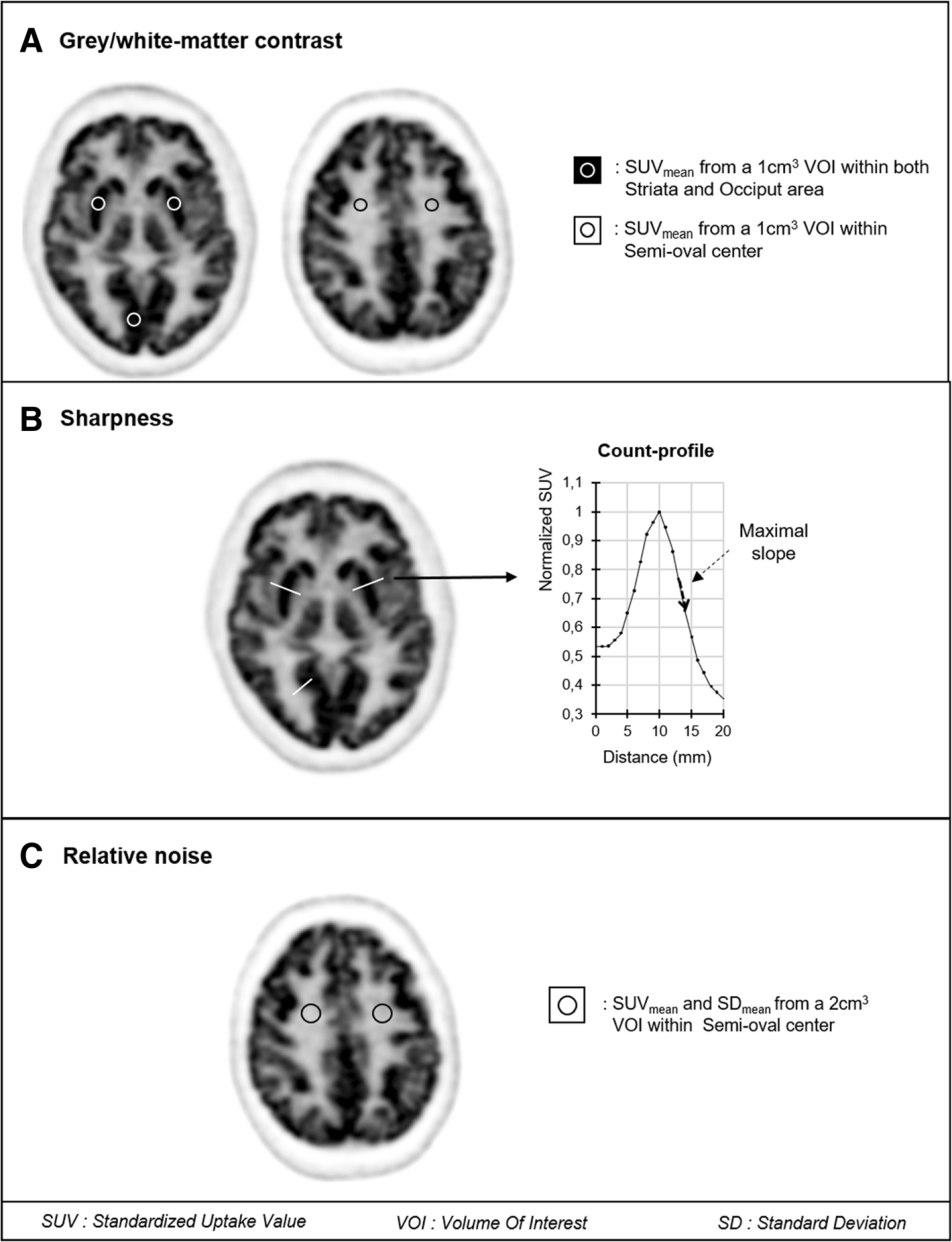
Schematic representations of methods used on axial ^18^F-FDG brain PET images to **a** quantify the contrast between central (striata) or peripheral (occiput) gray-matter structures and a white matter structure (semi-oval center), **b** determine the sharpness index through the maximal slope of count profiles obtained perpendicularly to the gray/white-matter interfaces of striata and occiput and further normalized to the maximal curve value, and **c** quantify the noise level within the semi-oval area

As detailed in Fig. 2a, *gray/white-matter contrast* values [13] were determined for both peripheral (occiput) and central gray-matter structures (averaged left and right striata values), relative to the white-matter semi-oval center, with 1-cm^3^ volume of interests (VOI) and according to the following formula involving mean standardized uptake values (SUV_mean_) for gray and white matter:

$$ \mathrm{Grey}/\mathrm{white}-\mathrm{matter}\ \mathrm{contrast}=\frac{{\mathrm{SUV}}_{\mathrm{mean}}^{\mathrm{grey}-\mathrm{matter}}-{\mathrm{SUV}}_{\mathrm{mean}}^{\mathrm{white}-\mathrm{matter}}}{{\mathrm{SUV}}_{\mathrm{mean}}^{\mathrm{white}-\mathrm{matter}}} $$

As detailed in Fig. 2b, *spatial resolution* was assessed through a sharpness index [14] computed as the maximum slope of count profiles. These count profiles were obtained perpendicularly to the gray/white-matter interfaces of the striata and occiput and are expressed in percent of the maximal count values and per millimeter length. Values from the 2 striata were averaged for further analyses.

*Relative noise* was assessed through a coefficient of variation (standard deviation-to-mean ratio) of the SUV from a 2-cm^3^ spherical VOI placed within the white-matter semi-oval center (see Fig. 2c). A VOI having a volume of 2 cm^3^ represents approximately the largest VOI that can be placed in the homogeneous zone of the semi-oval center.

### Visual analysis of image quality

A consensual visual assessment of 3 image quality parameters, i.e., contrast, spatial resolution, and noise, was obtained from two experienced physicians (AV, MP) on the 30 sets of FDG PET images without PSF correction (10 from analog PET with 2-mm voxels, 10 from digital PET with 2-mm voxels, and 10 from digital PET with 1-mm voxels). These image sets were rendered anonymous and presented in random order, and each image set was graded visually and subjectively with a 3-point scale, ranging from 1 to 3 for each of the 3 image quality parameters (from 1, the lowest class of quality score for contrast and spatial resolution but also the lowest class for noise level, to 3, the highest class of quality score for contrast and spatial resolution but also the highest class for noise level). Results of this visual analysis were presented through the sum-scores of the 10 sets of patient images for each reconstruction and for each image quality parameter (i.e., with a 30-point scale).

### Statistical analysis

Quantitative variables, expressed as median values and interquartile ranges due to non-normality distributions, were compared with the Mann-Whitney or Kruskal-Wallis test for 2-group or more-than-2-group comparisons, respectively. Paired comparisons of reconstructions obtained without or with PSF were performed using the Wilcoxon signed-rank test. A *p* value < 0.05 was considered significant.

## Results

The two patient groups, respectively investigated with analog and digital PET cameras, were each comprised of 5 women and 5 men and were similar in terms of age (63 [58–67] years vs. 58 [55–64] years), body mass index (24 [22–27] kg m^−2^ vs. 29 [25–31] kg m^−2^), and blood glucose (0.90 [0.88–0.94] g/L vs. 0.91 [0.87–0.98] g/L).

**Fig. 3:**
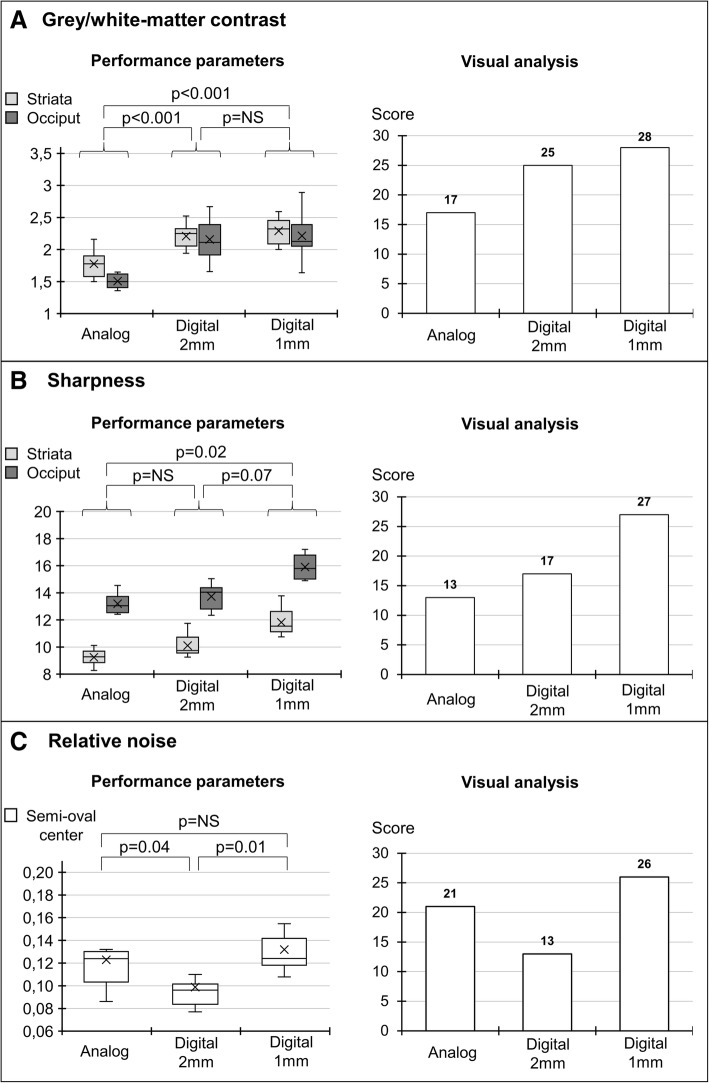
Left panels: box-plots for **a** gray/white-matter contrast, **b** sharpness, and **c** relative noise index obtained with current 2-mm reconstruction processes of analog and digital PET images and with a 1-mm reconstruction process developed for digital PET images. Right panel: corresponding parameters provided by a visual analysis from experienced physicians (sum-scores for contrast, spatial resolution, and noise level)

As detailed in Fig. 3 and in accordance with the corresponding results from visual analysis, most of the quantitative parameters of image quality were enhanced with digital PET reconstructed with a conventional 2-mm voxel size, as compared with analog PET. This enhancement was particularly pronounced for gray/white-matter contrast (27% increase for the median value of the striata and 41% for that of the occiput, *p* < 0.001, Fig. 3a), as well as for relative noise (22% decrease in median value (*p* = 0.04), Fig. 3c). A trend toward a better sharpness index was also documented for the same digital PET images (Fig. 3b) although this difference did not reach statistical significance.

However, the sharpness index was further enhanced for digital PET images reconstructed with the 1-mm voxel size, leading to reach a significant difference relative to the conventional images from analog PET (median increases of 24% for the striata and 21% for the occiput (*p* = 0.02), Fig. 3b), whereas the gray/white-matter contrast was unchanged, remaining higher than that of analog PET (median increases of 31% for the striata and 42% for the occiput (*p* < 0.001), Fig. 3a). This digital PET reconstruction with 1-mm voxel size was nevertheless associated with an increase in image noise, reaching a comparable level to that of analog PET (Fig. 3c).

**Fig. 4:**
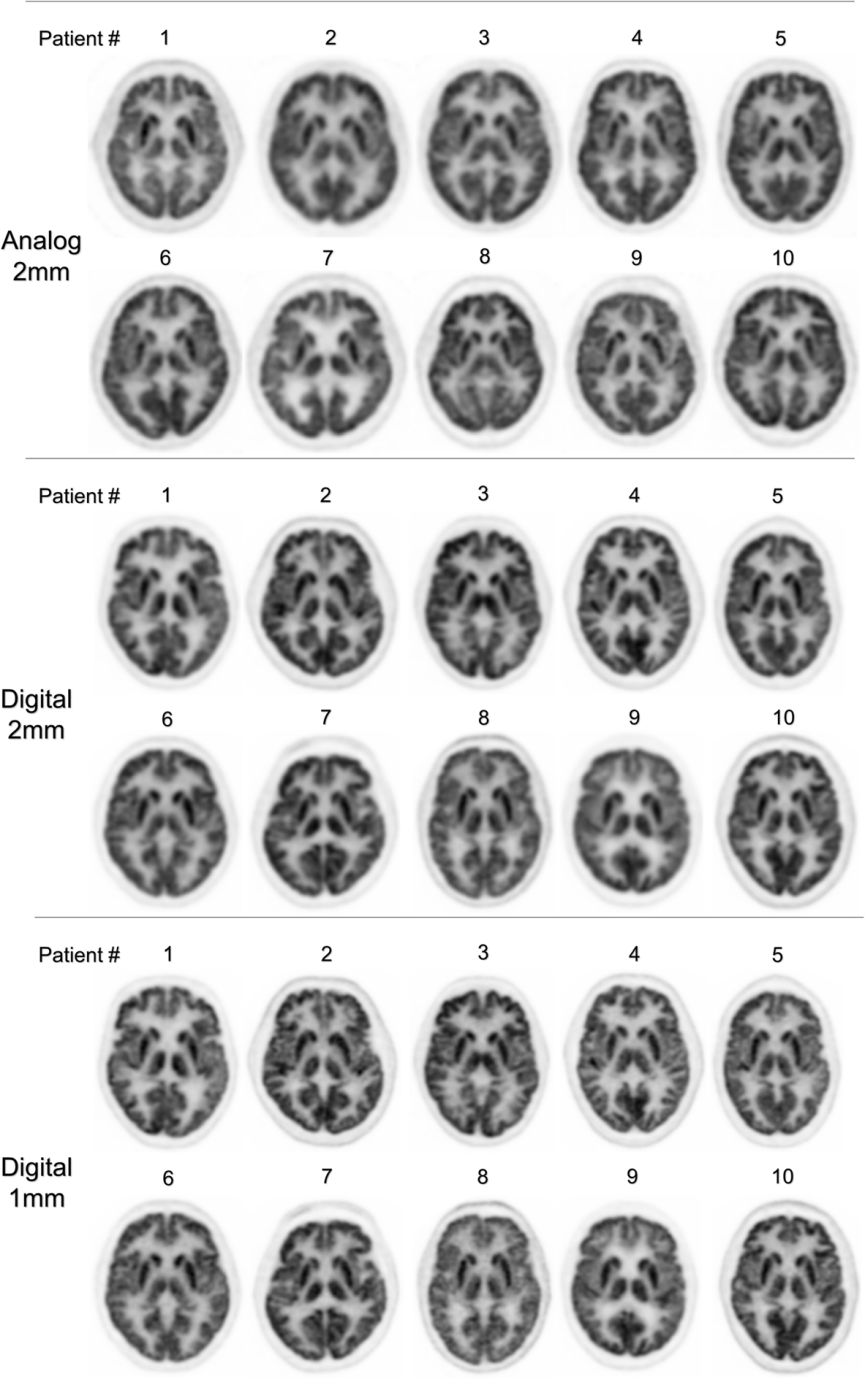
Gallery of axial brain ^18^F-FDG PET images obtained with analog PET and a current reconstruction method using a 2-mm voxel size (*n* = 10) (upper row), with digital PET and either a comparable reconstruction method with 2-mm voxel size (*n* = 10) (middle row) or a high-resolution reconstruction method with 1-mm voxel size (*n* = 10) (lower row)

A gallery of axial slices, extracted from all analyzed sets of PET images, is depicted in Fig. 4.

As detailed in the Additional file 1, all of the aforementioned differences remained significant when adding PSF deconvolution after the image reconstruction processes. This addition led to a systematic improvement in image contrast and spatial resolution and to an increase in image noise for all analyzed sets of analog and digital PET images (all *p* < 0.001).

Finally, as depicted in Fig. 3, the results from the subjective visual analysis of image contrast, noise, and spatial resolution were strongly comparable to those from the corresponding quantitative parameters, strengthening the consideration that the aforementioned differences in these quantitative parameters are sufficiently high to be clearly noticeable through a simple visual analysis by experienced physicians.

## Discussion

The present study shows that the image quality of normal brain ^18^F FDG PET images is improved with a fully digital PET, when compared with a last-generation analog PET camera and through an objective analysis of quantitative parameters. These improvements were particularly pronounced for image contrast but additionally for either image noise or spatial resolution, depending on the voxel size of the digital PET images (2 vs. 1 mm).

Furthermore, all of these improvements were sufficiently perceptible to be easily noticeable through a subjective visual analysis by experienced physicians, as illustrated in Fig. 3, and have thus the potential to enhance the routine examination of brain ^18^F-FDG PET scans.

The analysis of brain ^18^F-FDG PET images mainly relies on gray/white-matter contrast, the latter of which was found to be markedly enhanced with digital compared to analog PET, irrespective of the voxel size used for digital PET reconstruction. This contrast enhancement, reaching as high as 41% for the occiput, is of similar range to that documented between the same cameras for contrast recovery coefficients obtained from 10-mm-diameter hot spheres from the NEMA phantom [4, 5].

The amount of noise was also found to be significantly enhanced for digital PET with a greater than 20% noise reduction when using a conventional 2-mm voxel size reconstruction.

The enhancement in spatial resolution was however found to be rather poor for digital PET reconstructed with the routine 2-mm voxel size, although this resolution was clearly higher when voxels were reduced to a 1-mm size. These observations were strengthened through further experiments in which full-width at half maximum (FWHM) values were measured on a point source of F-18 according to the NEMA standard and thus, with filtered back projection reconstruction (experiments not shown). The 2- to 1-mm reduction in voxel size was associated with a decrease in FWHM of 15% for Vereos (from 4.73 mm to 4.0 mm at the center of field-of-view) and a much more limited change of 7% for Ingenuity (from 5.10 mm to 4.75 mm) [15]. Thus, it would appear that a 1-mm voxel size is more suited than the more conventional 2-mm size for taking advantage of the gain in spatial resolution which can be achieved with the Vereos. Such finding suggests that the voxel size should be adapted to the level of spatial resolution that can now be achieved with recent digital PET cameras.

The use of digital PET images with a 1-mm voxel size, as opposed to the more conventional 2-mm size, was not only associated with a high level of spatial resolution but also with a high image contrast, which remained higher than that of analog PET. These properties ultimately led to a very accurate delineation of the gray-matter structures, as evidenced in the images displayed in Fig. 4, reaching a level much closer to that currently obtained with MRI [16]. However, the drawback is a loss of advantage in terms of noise level, and therefore the clinical usefulness of such high-definition images remains to be fully investigated.

Of note, all of the above differences between digital and analog PET performances were independent of the use of PSF deconvolution recommended by the manufacturer. This deconvolution allows reducing the partial volume effect and therefore offers further improvements in image contrast and spatial resolution. However, noise is also amplified by the PSF deconvolution and, similarly to the choice of voxel size, a compromise is necessary between the levels of resolution and noise that need to be achieved for diagnostic purposes.

It should be emphasized that these differences in image contrast, noise, and spatial resolution were documented through optimization of the reconstruction processes in order to reach a high level of convergence for the contrast recovery coefficient for each camera and according to voxel size. This level of convergence was reached with a much lower number of iterations with the digital PET as opposed to the analog PET as evidenced in Fig. 1, a current observation due to the higher temporal resolution of the digital PET camera [17, 18].

A limitation of this study is that no 1-mm reconstruction software has been developed for routine examinations performed with the Ingenuity PET camera. Hence, no direct comparison could be performed between the 2 cameras for 1-mm voxel size images. However, it is likely that the higher levels of contrast and spatial resolution observed for digital PET on the present 2-mm brain images constitute a more favorable setting for analyzing PET images reconstructed through a 1-mm voxel size. The sample of patients (*n* = 10) used in the present study in both the analog and digital PET systems is limited, even if only patients with normal ^18^F-FDG PET brain images were retrospectively selected. Further studies with larger sample sizes should be conducted to confirm these preliminary results.

## Conclusions

On normal brain ^18^F-FDG PET images and compared with last-generation analog PET, fully digital PET offers clear improvements in contrast and image noise as well as a significant potential for further enhancing spatial resolution. These improvements are sufficiently noteworthy to be clearly noticeable visually and could be particularly appropriate in the setting of PET neuroimaging, by facilitating the delineation of cortical gyri in combination with MRI analysis.

## Additional file

Additional file 1: Box-plots for (A) grey/white-matter contrast, (B) sharpness and (C) relative noise index obtained with current 2-mm reconstruction processes of analog and digital PET images and with a 1-mm reconstruction process developed for digital PET images. Left panel without PSF deconvolution and right panel with PSF deconvolution. (TIF 2325 kb)

## Data Availability

The datasets used and/or analyzed during the current study are available from the corresponding author on reasonable request.

## Abbreviations

IEC: International Electrotechnical Commission
NEMA: National Electrical Manufacturers Association
PSF: Point spread function
TOF: Time of flight
