# Supplementary figures and images for: Head-to-head comparison of image quality between brain 18F-FDG images recorded with a fully digital versus a last-generation analog PET camera
Source: EJNMMI Res. 2019 Jul 12;9:61. doi: 10.1186/s13550-019-0526-5 (PMC6626093; doi:10.1186/s13550-019-0526-5)

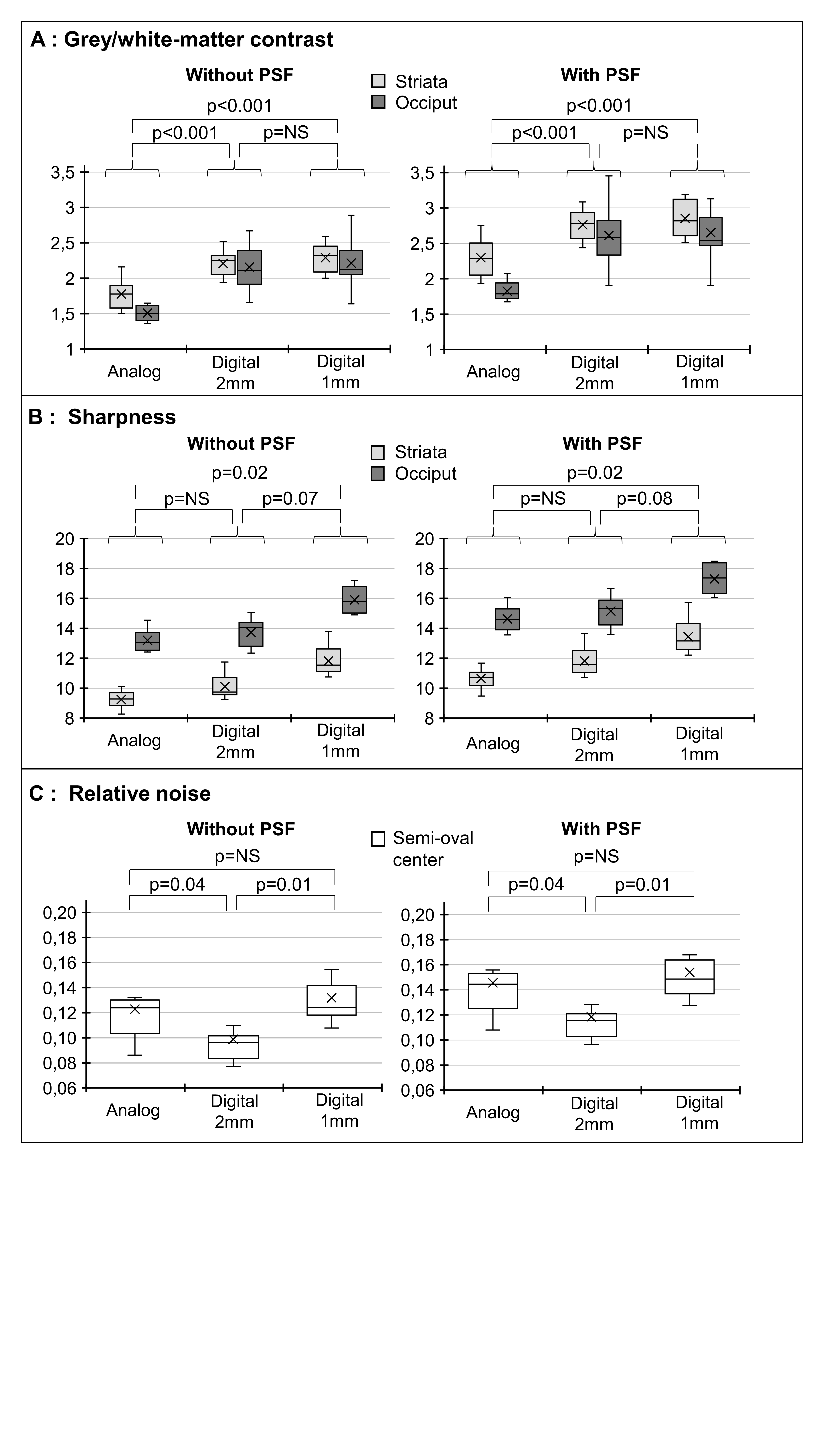

Supplement: Supplementary file 1 — Box-plots for (A) grey/white-matter contrast, (B) sharpness and (C) relative noise index obtained with current 2-mm reconstruction processes of analog and digital PET images and with a 1-mm reconstruction process developed for digital PET images. Left panel without PSF deconvolution and right panel with PSF deconvolution. (TIF 2325 kb) [file 13550_2019_526_MOESM1_ESM.tif]
